# Inhibitory effects of the ultrasound-targeted microbubble destruction-mediated herpes simplex virus-thymidine kinase/ganciclovir system on ovarian cancer in mice

**DOI:** 10.3892/etm.2014.1877

**Published:** 2014-08-04

**Authors:** XIAN-LONG ZHOU, YU-LU SHI, XIONG LI

**Affiliations:** 1 Emergency Center, Zhongnan Hospital, Wuhan University, Wuhan, Hubei 430071, P.R. China; 2 Medical College of Wuhan University, Wuhan, Hubei 430072, P.R. China; 3 Department of Ultrasound, Zhongnan Hospital, Wuhan University, Wuhan, Hubei 430071, P.R. China

**Keywords:** ultrasound, gene therapy, ovarian cancer, herpes simplex virus-thymidine kinase, microbubbles

## Abstract

The aim of the present study was to investigate the effect of the ultrasound-targeted microbubble destruction mediated (UTMD) herpes simplex virus-thymidine kinase (HSV-TK) and ganciclovir (GCV) system on ovarian cancer (OC). This study was conducted between June and December 2012 in the Animal Biosafety Level III Laboratory of Wuhan University. Mice with OC were randomly divided into four groups: i) HSV-TK plus microbubbles (MBs) plus ultrasound (US) (n=15); ii) HSV-TK plus US (n=15); iii) HSV-TK (n=15); and iv) phosphate-buffered saline (n=15). The inhibitory effect and survival time in the experimental groups were compared with those in the control group. The TK protein expression was detected by western blot analysis. Tumor cell apoptosis was detected by terminal deoxynucleotidyl transferase-mediated dUTP nick end labeling and caspase-3 activity analysis. The data showed that the efficiency of HSV-TK gene transfection and the tumor inhibitory effects were significantly increased in the HSV-TK plus MBs plus US group compared with those in the control group (P<0.01). UTMD-mediated HSV-TK treatment has also improved the rat survival rate (P<0.01). In conclusion, UTMD can effectively transfect the HSV-TK gene into target tissues and exert a significant inhibitory effect on OC in mice.

## Introduction

Ovarian cancer (OC) is the most common malignant tumor involving the ovaries and remains associated with a high mortality rate. Every year, ~191,000 cases are diagnosed worldwide (1). Despite advances in therapy, advanced OC maintains a five-year survival rate of ~40% (2). Thus, the necessity for novel therapeutic modalities exists (3,4). With the continuous development of molecular biology, gene therapy for OC has become a notable field of study (5). Suicide gene therapy is one of the most attractive tools. The most commonly used suicide gene approach uses herpes simplex virus-thymidine kinase (HSV-TK), which has been applied in several clinical trials (6,7). This transforms nucleoside analogs, such as ganciclovir (GCV), into monophosphorylated molecules, which are then converted into triphosphorylated forms by cellular enzymes. These triphosphorylated molecules are incorporated into elongated DNA, causing premature chain termination and cell death (8).

Despite gene therapy providing an alternative strategy for the prevention and treatment of cancer, its application in the clinic remains limited, primarily due to the lack of a safe and efficient gene delivery system (9). While viral and other classical vectors are currently favored, non-viral delivery systems may offer certain advantages for the delivery of therapeutic genes of interest. Non-viral delivery systems that are utilized in gene therapy for OC include injecting naked DNA, liposomes, polyplexes, lipopolyplexes and nanoparticles, as well as gene gun and ultrasound (US)/microbubble (MB)-mediated gene delivery (10). The injection of MBs into the blood, followed by the application of US, has been proved to be safe for humans (11). The theory behind the use of MBs as gene ‘vectors’ is that a focused US beam can be used to destroy the DNA-loaded MBs during their transit through the microvascular circulation, leading to localized transduction once the MB shell has been destroyed, while sparing non-targeted areas. US-targeted MB destruction (UTMD) has been used to deliver genes to cells *in vitro* and *in vivo* to treat diabetes, cardiovascular disease, lung cancer, thyroid cancer and even tendon diseases in experimental animal models (12–14). In the present study, a mouse OC model was used to investigate the potential inhibitory effects of the UTMD-mediated HSV-TK/GCV system on OC in mice.

## Materials and methods

### Preparation of plasmid and MBs

The pORF-HSV-TK plasmid (Auragene Bioscience, Changsha, China) was used in a polymerase chain reaction amplification with upstream (GAATTCATGGCCTCGTACCCCGGC) and downstream (CTCGAGTCAGTTAGCCTCCCCCATC) HSV-TK primers to obtain ~1.2 kb of the target HSV-TK fragment. The HSV-TK target gene was then connected to a pMD-18T simple vector (Takara Biotechnology, Co., Ltd., Dalian, China) to obtain the recombinant plasmid pMD-18T-TK. The recombinant plasmid was transformed into DH5a *Escherichia coli* competent cells (Auragene Bioscience) and spread on a lysogeny broth agar plate for 16 h of culture. The sequence was confirmed by the Beijing Genomics Institute (Beijing, China). The positive clones were then selected for plasmid extraction. The SonoVue^®^ MB contrast agent SF6 was purchased from Bracco (Milan, Italy); 90% of the MB diameters were measured to be <6 μm. The average diameter of the MBs was 2.5 μm. The gene-loaded lipid MBs were prepared as described in the study by Wang *et al* (15). The MBs were cultured with poly-L-lysine (1 mg/ml) (Sigma, St. Louis, MO, USA) at 37°C for 30 min. The subnatant was soaked, removed and washed twice with phosphate-buffered saline (PBS). Naked plasmid (1 mg/ml) was added and incubated at 37°C for 30 min, and washed twice using PBS. This manipulation was repeated three times. The plasmid concentration was measured to be 0.1 μg/μl.

### Animal models

The animal experiments were performed in the Center for Animal Experiments/Animal Biosafety Level III Laboratory of Wuhan University (Wuhan, China), and were conducted in accordance with the Guidelines for the Care and Use of Laboratory Animals of Wuhan University. Eighty female BALB/c-nu mice, aged six weeks and with a body weight of 14–16 g, were purchased from the Hubei Center for Disease Control and Prevention (Wuhan, China). Mice were raised in specific-pathogen free rooms under controlled conditions with a temperature of 23±3°C and relative humidity of 50±20%. Each BALB/c-nu mouse was inoculated with 2×10^6^ cells/site OC cells (SKOV3 cells, 10^7^ cells/ml, presented by the Medical Experimental Center of Zhongnan Hospital, Wuhan, China) subcutaneously into the right armpit under isofluorane anesthesia. In this model, tumors typically grew to 0.2 cm^3^ by the 15th to 20th day after injection. Sixty mice were then selected and randomly divided into four groups: i) HSV-TK plus MBs plus US (n=15); ii) HSV-TK plus US (n=15); iii) HSV-TK (n=15); and iv) PBS (n=15). Ten mice in each group were selected for a 14-day survival rate observation.

### US-assisted HSV-TK gene transfection in vivo

**Figure 1:**
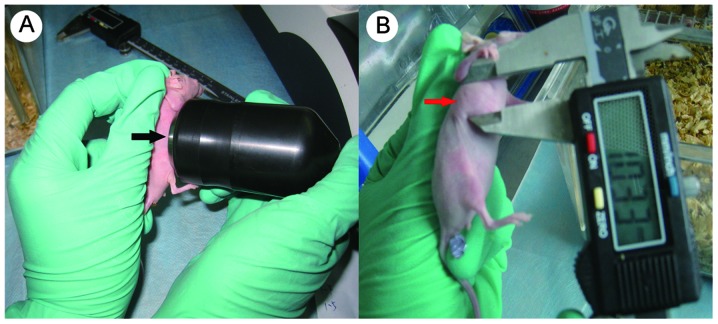
Gene transfection and tumor measurement. (A) Gene transfection following ultrasound irradiation. The ultrasound probe was tightly placed on the tumor mass (black arrow) to ensure that the whole tumor was exposed in the ultrasound. The ultrasound frequency was 1 MHz and sound intensity was 2 W/cm^2^. The pulse irradiation method was used for 5 min, with an interval time of 10 sec. (B) Tumor mass measurement. The red arrow indicates the tumor mass.

The mixture of plasmid and MBs was injected through the tail vein under isofluorane anesthesia on day 0. Only the HSV-T + MBs + US group received injection of the mixture of MBs and plasmid. The mice were injected once every three days, three times in total. PBS (200 μl) was administered in the PBS group and HSV-TK (200 μl, 0.1 μg/μl plasmid) was administered in the other three groups. A US transducer (Siemens, Berlin, Germany) was applied on the HSV-TK plus MBs plus US and HSV-TK plus US groups for irradiation following the gene injection. The US probe was tightly placed on the tumor mass to ensure that the whole tumor was exposed to the US. The US frequency was 1 MHz and sound intensity was 2 W/cm^2^. The pulse irradiation method was used for 5 min, with an interval time of 10 sec, according to the method described in the study by Zhou *et al* (16) (Fig. 1). Each mouse was intraperitoneally injected with 0.2 ml (100 mg/kg/day) GCV (Roche, Basel, Switzerland) from 24 h after irradiation. The GCV injection was performed in the HSV-TK groups every 24 h lasting for two weeks. The animals were sacrificed 24 h after the last GCV injection and tissues were harvested for pathological and biochemical examinations.

### Evaluation of tumor inhibitory effects

The maximum and minimum diameters of the tumor mass were measured with a digital caliper every two days. The size of the tumors was measured twice using the same method by two different experimenters. Tumor volume and inhibition rate (IR) were calculated on day 14 using the following formulae: Tumor volume (mm^3^)=(a × b^2^)/2, where ‘a’ refers to the maximum diameter (mm) and ‘b’ refers to the minimum diameter (mm); and IR = (average tumor size in the control group - mean tumor volume in the treatment group)/mean tumor volume in the control group × 100%. The growth curve was drawn according to the size of the tumor volume. The weight of the mice was also noted for the evaluation of their condition.

### Detection of apoptotic cells

Caspase-3 protease activity in the tumor tissue was measured using a caspase-3 colorimetric assay kit according to the manufacturer’s instructions (Beyotime Institute of Biotechnology, Shanghai, China). Briefly, the homogenates of the liver tissues were centrifuged, and protein (1 g) was incubated with Asp-Glu-Val-Asp-p-nitroanilide and reaction buffer for 90 min at 37°C. Absorbance measured at 405 nm was representative of caspase-3 activity. A terminal deoxynucleotidyl transferase-mediated dUTP nick end labeling (TUNEL) assay was carried out using a commercial kit (Roche Diagnostics GmbH, Mannheim, Germany). Apoptotic cells were identified by a brown stain over the nucleus. A total of ~200 cells were counted per field, five fields were examined per slide and five slides were examined per group. The apoptotic index (AI) was calculated as follows: AI = (number of apoptotic cells/total number of tumor cells) × 100%.

### Western blot analysis

Proteins were extracted using a protein extraction reagent (Sigma), following the manufacturer’s instructions. The TK protein was detected by western blotting. Concentrated gel (40 ml/l), separation gel (100 ml/l), pre-stained protein marker (3.0 μl) and sample total proteins (50 μg/hole) were prepared. The samples were added into 100 ml/l SDS for SDS-PAGE. The gel was removed when the bromophenol blue ran to the bottom, and the protein was synchronously transferred to the polyvinylidene difluoride membrane at 20 V for 50 min. This was then sealed for 4 h with 50 ml/l skimmed milk and Tris-buffered saline with Tween 20 (TBST) at room temperature (RT) following the trans-membrane procedure. The primary antibody was subsequently added (TK1 polyclonal antibody, 1:500; Abcam, Cambridge, UK) prior to incubation for 2 h at RT and maintenance overnight at 4°C. TBST was used to wash the membrane four times for 10 min/time. The horseradish peroxidase-conjugated secondary antibody (rabbit anti-rat antibody, 1:5,000; Boster Immunoleader, Wuhan, China) was then added for incubation, followed by agitation at RT for 2 h, washing of the membrane, imaging and exposure. The protein bands were normalized to GAPDH, and all blots were quantified with Quantity One software (Bio-Rad, Hercules, CA, USA).

### Statistical analysis

Experimental data are presented as the mean ± standard deviation. The data were processed by the statistical analysis software SPSS version 16.0 (SPSS Inc., Chicago, IL, USA). The analysis of variance was used to assess the IR. The Kaplan-Meier method was applied for the survival analysis. P<0.05 was considered to indicate a statistically significant difference.

## Results

### Therapeutic effects in vivo

**Figure 2:**
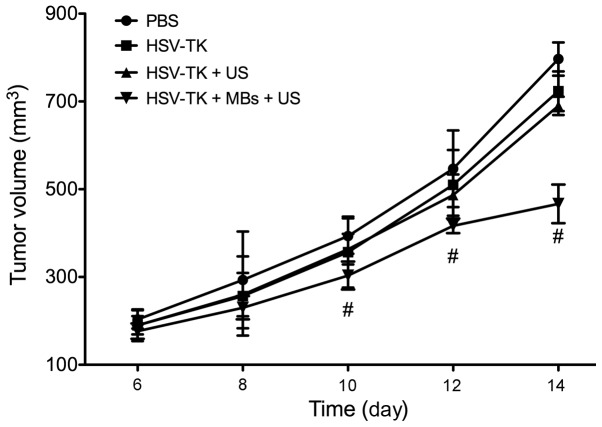
Tumor growth curve. The tumor growth rate in the HSV-TK + MBs + US group was significantly reduced compared with that in the other three groups. No significant difference was identified between the HSV-TK + US and HSV-TK groups. Data are presented as the mean ± standard deviation (n=10 in each group). ^#^P<0.01 compared with the other groups. PBS, phosphate-buffered saline; HSV-TK, herpes simplex virus-thymidine kinase; US, ultrasound; MB, microbubble.

**Figure 3:**
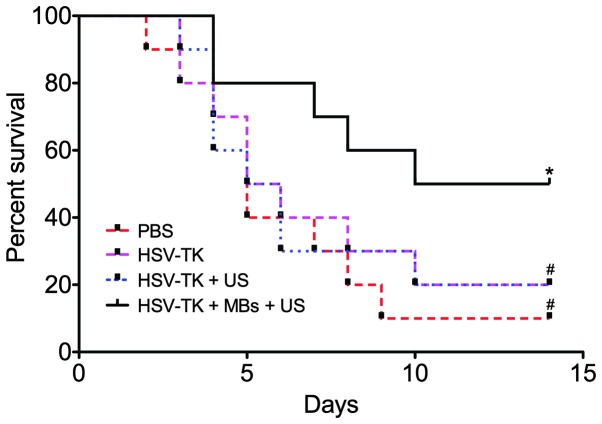
Rate of survival over 14 days (n=10 in each group). Compared with the HSV-TK + US and HSV-TK groups, the HSV-TK + MBs + US group showed a significantly higher survival rate during the observation period. ^#^P<0.01 compared with the HSV-TK + MBs + US group; ^*^P<0.01 compared with the PBS group. PBS, phosphate-buffered saline; HSV-TK, herpes simplex virus-thymidine kinase; US, ultrasound; MB, microbubble.

As the tumor size increased, the mice exhibited evident appetite loss, activity reduction, body weight loss and humpback posture. From the tumor growth curve, it was observed that the tumor growth in the HSV-TK plus MBs plus US group slowed significantly. Compared with the tumor sizes of the PBS group, the tumor sizes of the HSV-TK plus MBs plus US group were significantly lower during the last four days of observation (P<0.01) (Fig. 2). The tumor IRs of the HSV-TK plus MBs plus US, HSV-TK plus US, HSV-TK and PBS groups were 40.23±10.28, 22.20±3.73, 15.34±3.77 and 0%, respectively. In addition, it was observed that the tumor mass in the HSV-TK plus MBs plus US group was more easily separated from the normal tissue, was surrounded by fluid at the time of separation and exhibited a smooth surface. However, the tumors in the control group were difficult to separate without damaging the surrounding skin and tissues. The survival rate observation results showed that the HSV-TK plus MBs plus US group had a significantly higher survival rate (P<0.01) compared with the control group (Fig. 3).

### Apoptosis

**Figure 4:**
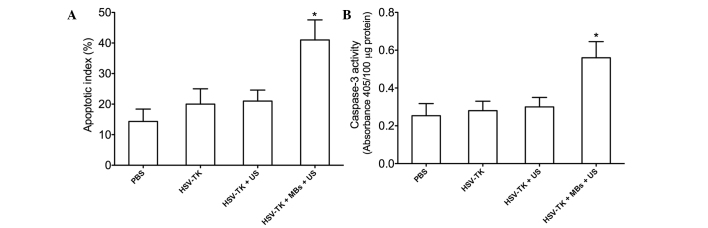
Apoptosis index and caspase-3 activity. (A) Apoptosis index of tumor cells; (B) caspase-3 activity in tumor tissues. The apoptosis of tumor cells in the HSV-TK + MBs + US group was significantly enhanced, as shown by a higher apoptosis index and caspase-3 activity level. Data are presented as the mean ± standard deviation (n=5 in each group).^*^P<0.01 compared with the other groups. PBS, phosphate-buffered saline; HSV-TK, herpes simplex virus-thymidine kinase; US, ultrasound; MB, microbubble.

TUNEL staining was performed to detect tumor cell apoptosis in each group. The TUNEL-positive cells (apoptotic cells) were stained brown. The tumor cells in each group underwent apoptosis to different degrees. The tumor cell apoptosis in the HSV-TK plus MBs plus US group was the most evident. The AI was highest (42.20±5.68%) in the HSV-TK plus MBs plus US group, which was significantly higher than that in the HSV-TK plus US (22.52±3.12%, P<0.01), HSV-TK (20.20±4.52%, P<0.01) and PBS (14.65±3.88%, P<0.01) groups (Fig. 4A). In addition, the caspase-3 activity in the HSV-TK plus MBs plus US group was significantly higher than that in the other groups (P<0.05, respectively) (Fig. 4B).

### HSV-TK gene transfer in vivo

**Figure 5:**
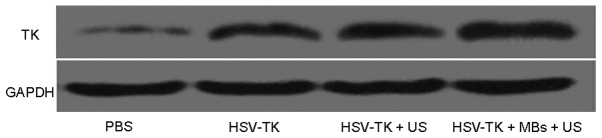
The TK protein in tumor tissues detected by western blot analysis. Each group has a single band at 25 kDa. The band intensity of the HSV-TK + MBs + US group was significantly higher than that in the HSV-TK + US and HSV-TK groups (0.65±0.20 vs. 0.20±0.05 and 0.18±0.04, respectively, P<0.01). TK, thymidine kinase; PBS, phosphate-buffered saline; HSV-TK, herpes simplex virus-TK; US, ultrasound; MB, microbubble.

The TK protein expression was detected in tumor tissues by western blot analysis. It was observed that a single band appeared in each group at 25 kd. The band in the HSV-TK plus MBs plus US group was the most evident (band intensity, 0.65±0.20), characterized by a higher band intensity (P<0.01) (Fig. 5). The bands in the HSV-TK plus US and HSV-TK groups were similar (band intensity, 0.20±0.05 and 0.18±0.04, respectively). However, no evident TK protein expression was identified in the major organs, including the heart, lung, liver and kidneys (data not shown).

## Discussion

OC is the leading cause of mortality from gynecological malignancies. Despite the fact that a response to initial therapy is exhibited by the majority of patients presenting with advanced disease, only 10–15% maintain a complete response following first-line therapy (17). Thus, using the enhanced understanding of the genetics and associated molecular pathways in OC, novel targeted drugs with a greater therapeutic specificity than standard chemotherapy are being developed (18). Different non-viral and viral vectors utilized for improvements in targeted therapy have been presented (3). In this study, UTMD was used to deliver plasmid DNA into target cells. UTMD can focus on specific tissues or organs directly and is beneficial for the targeted delivery of the gene of interest (19). Thus, a targeted and sustained antitumor effect is obtained. Furthermore, similar to non-viral vectors, MBs have a number of advantages over viral systems, particularly from the perspective of safety.

In the present study, western blot analysis was performed to confirm the TK expression, and it was revealed that UTMD could directly deliver the TK suicide gene to OC cells in this mouse model. The results of the western blot analysis showed that there was no evident TK protein expression in the major organs, including the heart, lung, liver and kidneys, which meant that the transfection of the TK gene was confined within the tumor cells. The mechanisms underlying UTMD-mediated gene transfer remain incompletely understood. The liposome MB is composed of polyethylene glycol and liposome, and the mean diameter of the liposome MB is smaller than that of red blood cells or conventional MBs; thus the liposome MB could be developed as a gene transfection tool and delivered into specific tissues (20). MB disruption at the US frequencies used in the present study can produce high-velocity fragmentation of the MB shell, which may contribute to gene transfection (21,22). This suggests that microporation of the vessel wall and/or the enhancement of cell membrane permeability may be responsible for transfection by UTMD.

A clear inhibitory effect on OC was observed in the present study. Compared with the other groups, tumor cell apoptosis in the HSV-TK plus MBs plus US group was more apparent. In addition, the tumor growth was significantly inhibited in the HSV-TK plus MBs plus US group. These findings established proof of concept that UTMD can impact tumor biology. Furthermore, it was observed that mice in the HSV-TK + MBs + US treatment group showed improved appetite, enhanced activity and lower body weight loss during this study. HSV-TK gene delivery into tumor cells followed by treatment with the antiviral drug GCV is the most common experimental and clinical model for gene therapy. The TK metabolizes the GCV into a monophosphorylated form, which is subsequently converted to a toxic triphosphate molecule by other cellular kinases. This toxic GCV metabolite is then incorporated into the DNA of replicating cells, leading to premature strand termination, replication failure and cell death. This type of pro-drug activation is widely described as a ‘suicide’ gene therapy (6,23). As one of the driving forces of the suicide gene strategy, ‘bystander effects’ have also been identified as important mechanisms of cytotoxicity and could ensure destruction of more tumor cells than the number that is actually infected. Those cells that have not been transfected can be supplemented by bystander effects, resulting in an anti-tumor effect (24,25). Additionally, MBs play an important role in enhancing the effect of gene therapy. A previous study (21) demonstrated that plasmids on the MB surface could be shielded from digestion by blood DNAses, in contrast to naked plasmids which are quickly degraded by blood DNAses upon vascular administration.

In conclusion, UTMD was used as a gene delivery method in the treatment of mouse OC by HSV-TK/GCV. The anti-tumor efficacy of HSV-TK and the mouse survival rate were markedly improved following use of the UTMD-mediated HSK-TK/GCV system. The UTMD technology is expected to provide a novel strategy for targeted OC therapy.
